# The New Sydenham Society’s Publications for 1861

**Published:** 1862-06

**Authors:** C. F. Heywood

**Affiliations:** 26 West 20th Street, New York


### THE NEW SYDENHAM SOCIETY'S PUBLICATIONS FOR 1861.

The following works will (should nothing unforseen prevent;) be issued the current year :

I.—*A Year-book of Medicine and Surgery for* 1860. (Nearly ready.)

II.—The First Volume of *Casper's Forensic Medicine.* (In the press.)

III.—The Second and Concluding Volume of *Frerick's Clinical Account of Diseases of the Liver.* The second volume of this work has not yet been published in the original. By the courtesy of its author and publishers (who have supplied the sheets as printed off) the Council will be enabled to have the translation ready almost simultaneously with the appearance of the work itself.

IV.—A volume of Selected Monographs, comprising

1. *Czermak on the Practical Uses of the Laryngoscope.*—Numerous wood cuts.
2. *Schrœder Van Der Kolk on Atrophy of the Brain.* Four lithographs.
3. *Dusch on Diseases of the Cerebral Sinuses.*
4. *Esmarch on the Uses of Cold in Surgical Practice.*
6. *Radicke's Papers on the Application of Statistics to Medical Enquiries.*

V.—*A Second Fasciculus of the Atlas of Portraits of Skin Diseases,* comprising Plates from *Hebra,* illustrating *Psoriasis Diffusa, Ichthyosis, Lupus Serpiginosus, Alopecia Areata.*

In addition to the above the following works are also in preparation for the Society:

*Vogel and Neubauer on the Examination of the Urine:* a manual intended for the assistance of the practical physician. With lithographs and wood cuts. (The publication of this work has been deferred on account of the expected appearance of a new and much amended edition of the original.)

*A Year-book of Medicine and Surgery for* 1861.

*The Second Volume of Casper's Manual of Forensic Medicine.*

*Professor Donders on the Diseases of Accommodation of the Eye.*—With a preliminary Essay on Dioptrics of the Eye, by the author.

*Smellie's Midwifery.* Reprinted, with notes and preface, &c., bringing the work up to the present standard of knowledge; by Prof. Simpson of Edinburgh.

Members can at present be supplied with the Series for 1859. A Provisional List of those desirous to obtain them is, however, in formation, and a Third edition is printed.

A few copies of the Series for 1860 are still on hand.

The *First Fasciculus of the Atlas of Skin Diseases* is now in course of distribution, and it is hoped that it will be in the hands of all members of the Society for the past year within the next fortnight. The issue of this work completes the series for 1860.

The Society now numbers upwards of 3,200 members.

The subscription is Six Dollars annually; to be paid *in advance.* The best mode of sending money is by post-office to the Secretary.

C. F. Heywood, 26 West 20th Street, New York.

Note.—All connected with the profession, including students, are eligible as members: those who may wish to become such will save much trouble by promptly sending in their names to the Secretary.

